# Craniofacial and Dental Complications Following Total Glossectomy Without Multidisciplinary Follow-Up: A Case Report

**DOI:** 10.3390/dj13120559

**Published:** 2025-11-28

**Authors:** Fatima Salek, Loubna Bahije, Fatima Zaoui

**Affiliations:** Department of Orthopedic Dento Facial, Faculty of Dental Medicine of Rabat, University Mohamed V, Allal El Fassi Avenue, Mohamed Jazouli Street, Rabat 6212, Morocco; loubnabahije@gmail.com (L.B.); f.zaoui@um5s.net.ma (F.Z.)

**Keywords:** lymphangioma, tongue, case report, dental repercussions, interdisciplinary management

## Abstract

**Background**: Lymphangiomas are rare benign malformations of the lymphatic system, most frequently affecting the head and neck region. Oral involvement is uncommon, and when the tongue is affected, particularly its anterior two-thirds, it can cause major functional and esthetic disturbances. **Case Presentation**: We report the case of a 13-year-old boy who had undergone total glossectomy at the age of 4 for cystic lymphangioma. During follow-up, the patient presented marked dental and craniofacial alterations, including severe maxillary and mandibular crowding and a skeletal Class III pattern. This case highlights the significant morphological and functional consequences resulting from the absence of the tongue during growth. **Conclusions**: This case underscores the crucial role of the tongue in craniofacial growth and occlusal development. Early total glossectomy can result in long-term dental and skeletal disturbances, emphasizing the importance of early multidisciplinary follow-up combining surgical, orthodontic, and functional rehabilitation. Further studies are needed to establish appropriate management and rehabilitation protocols for such rare cases.

## 1. Introduction

Malocclusion of the teeth and dentofacial deformities generally result from the interaction between innate genetic factors and environmental influences. Environmental factors affect both jaw growth and the proper positioning of teeth within a functional equilibrium. Any disruption of this balance can lead to malocclusion and dentofacial deformities, impacting both esthetics and masticatory function [1].

The tongue, primarily composed of striated muscle tissue, plays a central role within the oral cavity. It contributes to essential functions such as sucking, chewing, swallowing, and speech, all of which are critical for maintaining a satisfactory quality of life [2]. Beyond its functional role, the tongue also exerts a significant morphogenetic influence on craniofacial growth. During the active growth phase, it helps maintain a dynamic equilibrium between soft tissue pressure and developing skeletal structures. Alterations in tongue mobility or volume—whether congenital or acquired, as in microglossia, aglossia, or hemihypertrophy—can disrupt the harmonious development of the maxillofacial skeleton [3].

Recent advances in three-dimensional (3D) imaging and morphometric analysis have provided new insights into craniofacial growth and reinforced the relationship between tongue posture, muscular balance, and craniofacial form (Hajeer et al., 2004; Chiou W-C et al., 2025) [4,5]. These findings confirm the tongue’s crucial role in guiding maxillary and dental development.

Lingual lymphangiomas are a rare cause of macroglossia in children, and in severe cases, may necessitate extensive tongue resection. They can interfere with feeding, speech, and airway function [6,7]. While lymphangiomas are uncommon, the clinical relevance of their management lies primarily in their functional and developmental consequences, rather than the pathology itself.

There are very few reports in the literature describing the management of patients who undergo extensive or total glossectomy, and none specifically address long-term craniofacial, dental, and functional outcomes. This lack of evidence represents a significant knowledge gap, particularly regarding the impact of early total glossectomy on jaw growth, oral function, and overall quality of life.

The present report describes a rare case of early total glossectomy performed at four years of age. This case is unique due to the combination of early age at surgery, absence of structured multidisciplinary follow-up, and severe craniofacial and dental sequelae. It highlights the importance of early diagnosis, close functional monitoring, and coordinated multidisciplinary management to minimize the long-term consequences associated with absence of the tongue during growth.

## 2. Case Presentation

### 2.1. Patient Background

A 13-year-old boy was referred to the Department of Orthodontics of Mohammed V University in Rabat, Faculty of Dental Medicine with the chief complaint of severe dental crowding in the maxillary and mandibular arches. The patient was born to healthy, non-consanguineous parents after an uncomplicated full-term pregnancy. Family history was unremarkable, with no relevant systemic conditions, and no other masses were found in the head and neck region. The patient’s medical history revealed that the condition began at age two, when swelling of the tongue affecting feeding and breathing was first observed. Despite several symptomatic treatments, no improvement occurred, and by age three and a half, the mother noted progression of the swelling. At four years old, the diagnosis of cystic lymphangioma of the tongue was made, and the patient underwent a total glossectomy.

### 2.2. Functional Examination

#### 2.2.1. Speech

Speech disturbances were assessed using the Frenchay Dysarthria Assessment (Enderby, 1983) [8]. Quantitative analysis revealed significantly impaired articulatory precision, with scores of 2/5 for alveolar sounds (/t/, /d/, /n/, /l/) and 2/5 for sibilants (/s/, /z/). Overall speech intelligibility was rated at 3/10, indicating severe reduction. The assessment was independently performed by two trained clinicians with 90% inter-rater agreement.

#### 2.2.2. Chewing

Masticatory function was evaluated using the Ono color-changeable chewing gum test (Tarkowska et al., 2017) [9]. The patient required 45 masticatory cycles to achieve bolus formation (norm: 15–20 cycles), with a color change score of 2/10 indicating severely reduced efficiency. Compensatory movements of the lips and cheeks were observed in 85% of chewing sequences, and the patient demonstrated preference for soft foods exclusively.

#### 2.2.3. Breathing

Nasal airflow assessment using the mirror fogging test (Roithmann, 2007) [10] showed minimal fogging (1/3) during quiet respiration, confirming predominant oral breathing. Lip seal at rest was incomplete, with an interlabial gap of 4 mm measured during quiet breathing.

#### 2.2.4. Swallowing

Swallowing function was evaluated using the Logemann Clinical Swallowing Evaluation Protocol (1985) [11]. With liquid textures, the patient exhibited compensatory head movements in 100% of swallows and required 2–3 attempts per bolus. For solid textures, the oral preparatory phase was significantly prolonged to 15–20 s, and oral residue was observed in the buccal and palatal spaces after 80% of swallows. These quantitative measures confirm severely compromised swallowing efficiency without tongue propulsion.

### 2.3. Extraoral Clinical Examination

**Figure 1:**
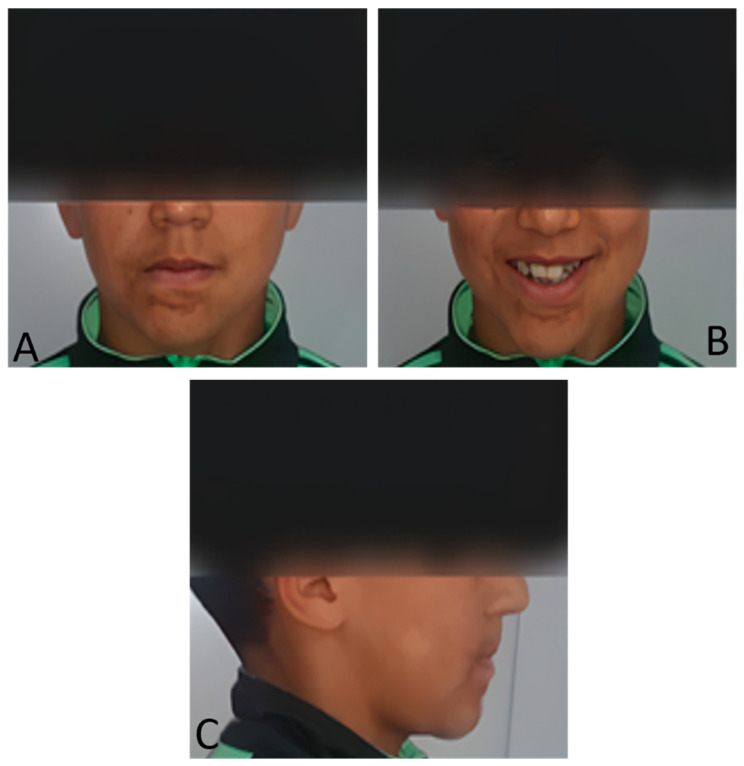
Extraoral photographs of the patient. (**A**) Frontal view showing a slight deviation of the chin to the right and an increased lower facial height giving the face an oval appearance and the presence of a subtle scar below the lower lip. (**B**) Smiling view illustrating a disharmonious smile with deviation of the upper interincisal midline to the right relative to the midsagittal plane. (**C**) Profile view revealing a convex subnasal profile with an open nasolabial angle. The lips appear hypertonic, reflecting compensatory muscular activity related to the absence of the tongue.

The extra oral examination (Figure 1) showed:

- A slight deviation of the chin to the right and an increase in the height of the lower part of the face give it a distinct oval appearance.
- Convex subnasal profile with an open nasolabial angle marked by a subtle scar under the lower lip.
- A disharmonious smile, with a deviation of the upper midline interincisal line to the right from the midsagittal plane.

The examination of the temporomandibular joint revealed no discrepancy between the centric relation and the centric occlusion, and the patient did not report any pain or clicking at the joint.

The dental arches further demonstrate the developmental impacts of early total glossectomy.

### 2.4. Intraoral Clinical Examination (Figure 2)

- Maxillary arch: Crowding of the anterior teeth and a distinctive V-shaped arch are associated with the absence of teeth 14 and 24. Notably, the buccal positions of teeth 13 and 23 and the lingual positions of teeth 12 and 22 demonstrate difficulty in finding space and maintaining good symmetry.
- Mandibular arch: Similar crowding and a V-shape in the mandibular arch are accompanied by the absence of teeth 31, 36, and 45, as well as poor positioning of the incisors, reinforcing the skeletal adaptations caused by the lack of lingual function.
- Interarch relationships: Class III canine and molar relationships characterize a reverse overjet between teeth 26 and 36, with both maxillary and mandibular arches showing endognathia, a hallmark of altered growth patterns.

**Figure 2:**
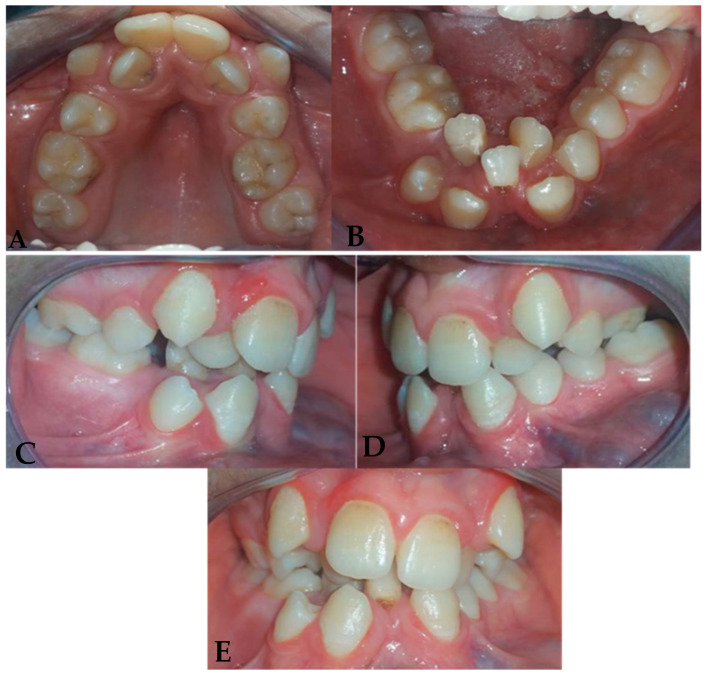
Intraoral photographs of the patient. (**A**) Maxillary occlusal view showing anterior crowding and a V-shaped arch form, associated with the absence of teeth 14 and 24. The buccal displacement of canines (13 and 23) and the lingual displacement of lateral incisors (12 and 22) highlight space deficiency and loss of arch symmetry. (**B**) Mandibular occlusal view revealing similar crowding and a V-shaped arch, with absence of teeth 31, 36, and 45. The irregular alignment of the incisors reflects adaptive skeletal and dental responses secondary to impaired lingual function. (**C**–**E**) Frontal and lateral intraoral views showing Class III canine and molar relationships, an anterior crossbite, and reduced overjet. Both maxillary and mandibular arches present transverse constriction (endognathia), consistent with altered craniofacial growth patterns following tongue resection.

### 2.5. Radiographic Examination

**Figure 3:**
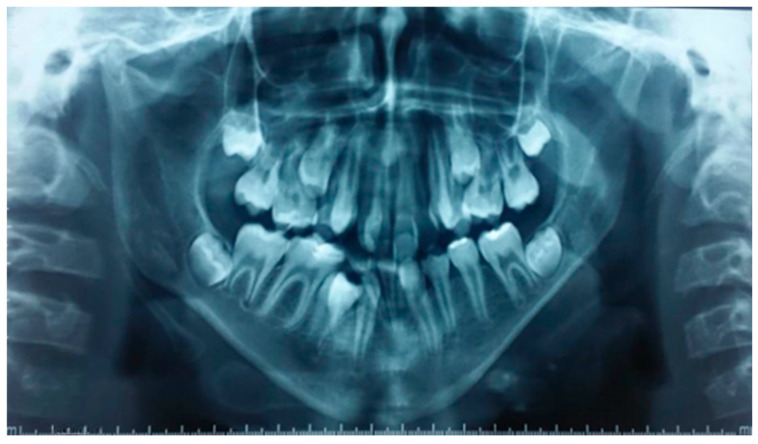
Panoramic radiograph of the patient. The panoramic view confirms the inclusion of teeth 15, 25, and 45, as well as the absence of tooth 36. The developing third molar germs are visible bilaterally. A noticeable skeletal asymmetry was observed between the right and left sides, with a thicker mandibular ramus and condylar region on the right compared to the left.

The panoramic radiograph (Figure 3) confirms the inclusion of teeth 15, 25, and 45, the absence of tooth 36, and the presence of the wisdom tooth germs.

**Figure 4:**
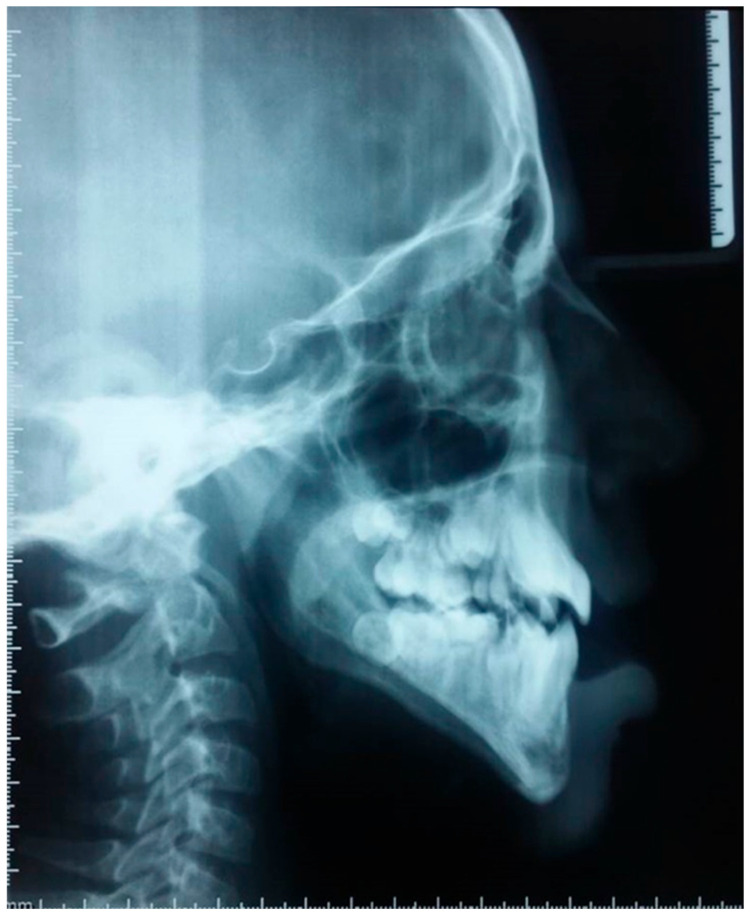
Lateral cephalometric radiograph. Cephalometric analysis revealed a complex pattern combining true mandibular retrognathia (decreased SNB) with a skeletal Class III anteroposterior discrepancy (negative AoBo). This apparent contradiction is characteristic of a severe hyperdivergent facial growth pattern with posterior mandibular rotation (GoGn/SN = 53°), a direct consequence of tongue absence and the disruption of muscular forces on craniofacial growth.

A lateral cephalometric radiograph was obtained (Figure 4), and the analysis is summarized in Table 1. The results reveal a severe and complex craniofacial dysmorphism directly linked to the glossectomy.

- Sagittal Skeletal Relationship: The SNA angle (70°) indicates significant maxillary retro position. The SNB angle (71°) confirms true mandibular retrognathia. Despite this retrognathic mandible, the ANB angle is on the lower end of normal (1°), and the AoBo measurement is severely negative (−7 mm). This combination unequivocally establishes a skeletal Class III relationship.
- Vertical Skeletal Relationship: The most striking finding is a severely hyperdivergent growth pattern, evidenced by the drastically increased GoGn-SN angle (53° compared to a norm of 32° ± 5).
- Dental Compensations: The incisor analysis revealed compensatory adaptations. The upper incisors are proclined (I/NA: 24°) and protruded (itoNA: 7 mm), while the lower incisors are retroclined (I/NB: 19°) and retruded (itoNB: 1 mm), classic mechanisms in skeletal Class III cases.

In summary, the cephalometric analysis confirms a triad of maxillary retroposition, mandibular retrognathia, and a hyperdivergent facial type, culminating in a skeletal Class III malocclusion. These findings are direct sequelae of the absent tongue, which has disrupted the normal functional matrix, leading to unopposed vertical growth and a lack of sagittal development. The absence of the tongue shadow was also visually confirmed on the radiographs (Figure 3 and Figure 4).

## 3. Discussion

The growth of the maxilla and mandible results from a complex interplay of genetic and environmental factors. While genes regulate craniofacial morphogenesis, they do not precisely dictate final morphology; rather, they modulate cellular responses to functional and environmental stimuli [12,13]. Consequently, craniofacial growth remains highly sensitive to mechanical and physiological influences.

Edward Angle’s 1907 observation that “… there are as many variations as there are cases met with, resulting and corresponding with variations in the malocclusion” remains relevant [14]. Contemporary studies confirm that oral dysfunctions—such as tongue thrusting, mouth breathing, or abnormal tongue posture—contribute to the development of malocclusions [6,12]. In our patient, the absence of a functional tongue following total glossectomy at age four caused profound disruption of orofacial equilibrium, illustrating the long-term skeletal and dental consequences of impaired tongue function during critical growth periods.

Lymphangiomas, although benign, can produce significant deformities when located on the tongue [15,16,17,18]. These lesions typically appear at birth or during early childhood [1,2]. In the present case, the lesion developed at four years of age and caused progressive macroglossia that interfered with feeding, speech, and airway patency, ultimately necessitating a total glossectomy. To our knowledge, no previously published case has described a complete glossectomy performed at such an early stage of craniofacial development. Most reports in the literature concern partial glossectomies or resections conducted in adults, in whom craniofacial growth is largely completed [19,20]. Compared with those cases, our patient’s early and total loss of tongue function appears to have induced a more severe bimaxillary endognathia and marked crowding, emphasizing the unique morpho functional consequences of total tongue absence during active growth.

Quantitatively, cephalometric analysis revealed marked skeletal discrepancies: the patient exhibited a reduced SNA angle (70° vs. 82° ± 2), a retrognathic mandible (SNB = 71° vs. 80° ± 2), and an extreme vertical pattern (GoGn–SN = 53° vs. 32° ± 5). These deviations of 4–6 standard deviations from normative values confirm a combined sagittal and vertical deficiency. These findings are consistent with, yet substantially exceed, the effects observed in experimental models. While Harvold’s (1968) [21]. partial glossectomy (2 cm wedge resection) in primates produced dental crowding and deep bite within 4–6 months, our case of total glossectomy resulted in far more severe skeletal consequences. The 15–20% reductions in mandibular length reported in animal studies [21,22] find their clinical correlate in our patient’s extreme cephalometric values. This case therefore provides rare human evidence supporting the critical role of the tongue in maintaining normal sagittal mandibular development and controlling vertical facial growth, demonstrating a clear dose–response relationship between the extent of tongue loss and the severity of craniofacial impairment.

The tongue plays a central role in maintaining neuromuscular balance within the oral cavity, influencing tooth positioning, mandibular arch dimensions, vertical facial height, and chin projection [18]. In the absence of lingual support, the perioral and buccinator muscles exert unopposed pressure, leading to arch constriction and crowding—findings that were quantitatively confirmed in our patient through transverse arch reduction and V-shaped morphology in both jaws.

Three-dimensional imaging techniques, as described by Hajeer et al. (2004) [4], can provide valuable information on the impact of soft-tissue changes on skeletal morphology and occlusal relationships. Although 3D imaging was not available for this patient, future cases could benefit from these tools to quantify soft-tissue and skeletal adaptations and guide interdisciplinary treatment planning.

The tongue also contributes significantly to mastication and swallowing, functions that shape maxillary and mandibular development [23,24]. The posterior tongue’s upward pressure during swallowing supports normal palatal growth; its absence may result in a narrowed palate, nasal obstruction, and oral breathing [3]. These compensatory adaptations—oral breathing, perioral muscle hyperactivity, and inefficient swallowing—were all evident in our patient, confirming the functional cascade initiated by the absence of lingual propulsion. Similar patterns of altered deglutition and airway function have been associated with skeletal Class III malocclusions in the works of Cayley et al. [25] and Fuhrmann and Diedrich [26]. However, in our case, the combination of glossectomy, oral breathing, and tooth loss likely acted synergistically to accentuate craniofacial imbalance, rather than resulting from tongue loss alone.

Furthermore, reduced tongue volume can limit alveolar development, resulting in insufficient space for tooth eruption and dental impaction [27]. In our patient, the early disturbance of eruption sequences and asymmetric tooth positioning may have further compounded the skeletal and dental discrepancies. These findings highlight the multifactorial etiology of the observed deformity, in which surgical, functional, and developmental factors interact dynamically.

Early rehabilitation, including both speech therapy and myofunctional exercises, is critical for restoring oral function and promoting balanced craniofacial growth [28]. The absence of structured postoperative follow-up between ages 4 and 13 likely exacerbated skeletal and functional discrepancies. Orthodontic management in this case played a pivotal role in reestablishing arch coordination and facial harmony, consistent with evidence indicating that early interceptive treatment can normalize growth trajectories and reduce the need for extensive surgical corrections [29,30].

In conclusion, this case demonstrates that total glossectomy during early childhood can lead to profound, quantifiable alterations in both sagittal and vertical craniofacial development. The absence of comparable cases in the literature reinforces the novelty and clinical relevance of these findings. Comprehensive, multidisciplinary care including surgery, speech therapy, and orthodontic intervention is essential not only for functional recovery but also for mitigating skeletal imbalance and improving psychosocial adaptation.

### Clinical Relevance

This case represents, to our knowledge, the first documented instance of total glossectomy performed at four years of age, offering unique insights into the long-term craniofacial and dental sequelae of early tongue loss. The quantitative cephalometric data provide objective evidence of severe sagittal and vertical underdevelopment, supporting experimental observations regarding the morphogenetic role of the tongue.

The report underscores the critical need for early, coordinated multidisciplinary management involving surgeons, orthodontists, and speech therapists to minimize functional and skeletal complications.

By comparing this case with previously reported partial glossectomies and experimental studies, it highlights how complete early tongue absence produces a distinct and more severe growth pattern, rarely documented in the literature.

## 4. Conclusions

Total glossectomy performed at an early age can profoundly alter craniofacial growth and oral function. This case emphasizes the tongue’s key morphogenetic role and highlights how its absence disrupts the balance of skeletal and dental development. Early and coordinated multidisciplinary management—including surgical, orthodontic, and speech therapy interventions—is essential to support functional adaptation and psychological well-being during growth.

However, the conclusions drawn from a single case must be interpreted with caution. The lack of longitudinal follow-up and three-dimensional assessment limits the ability to fully characterize growth dynamics. Future studies should aim to collect quantitative and functional data from larger cohorts to develop evidence-based rehabilitation protocols and improve the long-term quality of life for patients undergoing early glossectomy.

## Figures and Tables

**Table 1:** Cephalometric analysis.

Parameter	Patient	Norm	Interpretation
SNA	82° ± 2	70°	Maxillary retroposition
SNB	80° ± 2	71°	Mandibular retrognathia
ANB	2° ± 2	1°	Normal sagittal relationship
AoBo	0 ± 2	−7 mm	Skeletal Class III
GoGn/SN	32° ± 5	53°	Hyperdivergent vertical growth
I/NA	22°	24°	Upper incisor proclination
itoNA	4 mm	7 mm	Upper incisor protrusion
I/NB	25°	19°	Lower incisor retroclination
itoNB	4 mm	1 mm	Lower incisor retrusion

SNA: the maxilla (point A) is related to the cranial base (SN); SNB: the mandible (point B) is related to the cranial base (SN); AoBo: represents the projection of the points A and B on the occlusal plane; ANB: is the difference between SNA and SNB; GoGn/SN: angle formed by lines SN and GoGn; I/to NA (mm): maxillary incisor position; I/to NA: maxillary incisor version; i/ to NB (mm): lower incisor position; i/ to NB: lower incisor version; Po to NB: most anterior part of the symphysis of the mandible to line NB; GoGnSN: mandibular plane to cranial base.

## Data Availability

No new data were created or analyzed in this study. Data sharing is not applicable to this article.

## Abbreviations

The following abbreviations are used in this manuscript:

SNA	The maxilla (point A) is related to the cranial base (SN)
SNB	The mandible (point B) is related to the cranial base (SN)
AoBo	Represents the projection of the points A and B on the occlusal plane
ANB	Is the difference between SNA and SNB
I/to NA (mm)	Maxillary incisor position
I/to NA	Maxillary incisor version
i/ to NB (mm)	Lower incisor position
i/ to NB	Lower incisor version
Po to NB	Most anterior part of the symphysis of the mandible to line NB
GoGnSN	Mandibular plane to cranial base
